# Editorial: Neural mechanisms of motor planning in assisted voluntary movement

**DOI:** 10.3389/fnhum.2025.1582214

**Published:** 2025-03-13

**Authors:** Suriya Prakash Muthukrishnan, Adham Atyabi

**Affiliations:** ^1^Department of Physiology, All India Institute of Medical Sciences, New Delhi, India; ^2^Department of Computer Science, University of Colorado, Colorado Springs, CO, United States

**Keywords:** brain-machine interfaces, electroencephalography (EEG), error processing, functional near-infrared spectroscopy (fNIRS), kinematic synergies

Advancements in assistive robotics for motor rehabilitation have made significant progress in recent years, with Brain-Machine Interfaces (BMIs) playing a key role in decoding motor intentions and enhancing voluntary movement control. EEG-based systems are the most widely studied non-invasive BMIs, while fNIRS-based approaches are gaining interest (Lebedev and Nicolelis, 2017). Non-invasive BMIs, primarily explored in clinical studies, aim to promote neurorehabilitation by facilitating brain plasticity and motor recovery (Lebedev and Nicolelis, 2017). Research suggests BMIs could evolve beyond assistive technology into a therapeutic tool for neurological recovery (Donati et al., 2016). Effective BMIs integrate both top-down movement intention and bottom-up sensory feedback (Scott, 2016). This editorial highlights key contributions from five articles (four research articles, one review) featured in this Research Topic, addressing advances and challenges in human motor neuroscience and human-machine teaming, focusing on neurorehabilitation and the development of brain-machine interfaces for assistive robotics.

Improving humanoid robot hand dexterity enhances their ability to perform precise tasks, such as surgical assistance and aiding individuals with disabilities. The brain simplifies voluntary movement by organizing muscle and joint activations into coordinated patterns called “synergies,” reducing the complexity of controlling the hand's numerous degrees of freedom. Studying these synergies provides insights into brain-hand communication, motor disorders, and robotic control. Researchers have explored kinematic synergies (Grinyagin et al., 2005; Freitas et al., 2006), to better understand the neural activity that mediates musculoskeletal mechanics and behavioral goals. Olikkal et al. extracted kinematic synergies from 33 American Sign Language hand gestures using an RGB camera, MediaPipe, Gaussian functions, and Principal component analysis (PCA), achieving 95.7% accuracy. This synergy-based approach simplifies motion retargeting, offering potential for assistive robotics.

Accident analyses highlight run-off road incidents as a major cause of fatalities, yet drivers' brain responses during such events remain poorly understood. Brain recordings capture overlapping processes such as motor control, visual processing, and error monitoring, making their individual contributions unclear. While studies have separated visual and motor components (Walter et al., 2001; Horikawa et al., 2005; Garcia et al., 2017), disentangling these processes remains challenging. Pulferer et al. addressed this by employing passive and active steering in error-free and error-prone conditions, demonstrating that distinct sub-processes can be separated using time-locked analyses of EEG data. Findings revealed increased fronto-central activity and information flow during execution, linked to performance monitoring in the caudal anterior cingulate cortex.

Reaching movements, fundamental to daily life and rehabilitation, follow Fitts' Law, which predicts longer movement times for more difficult tasks (Fitts, 1954). While Fitts' Law has enhanced human-machine interactions in neurorehabilitative devices (Zimmerli et al., 2012), the association between cortical activation and task demands remains unclear, hindering optimal therapeutic parameter selection (e.g., dosage, repetition, difficulty). Ji et al. examined whether motor cortex activity correlates with index-of-difficulty in speed-accuracy reaching tasks. Healthy subjects performed 2D reaching movements with ID levels using a rehabilitation robot while fNIRS recorded cortical responses. While kinematic data aligned with Fitts' Law, motor cortex activity showed no direct correlation with task difficulty, implying the influence of additional factors such as muscle activation.

Physical exercise enhances brain plasticity, crucial for functional reorganization of the lesioned cortex and motor recovery in patients with motor impairment (Kokotilo et al., 2009; James and McGlinchey, 2022). However, how different movement patterns influence somatosensory cortex reorganization across various stages of neurorehabilitation remains poorly understood. Understanding their specific effects on sensory-motor cortex activity can refine training dosages (type, time, and intensity) and deepen our understanding of exercise mechanisms. To explore the impact of exercise training modes on sensory and motor-related cortex excitability, Li et al. used fNIRS to study cortical activity in healthy participants during passive, active, and resistance tasks with an upper-limb robotic device. Active movement showed higher contralateral M1 activation, while resistance exercise activated both hemispheres more extensively. While these findings provide valuable insights, further research is needed to refine exercise therapy strategies using assistive technologies.

Human movement is defined by kinematic and kinetic attributes. Kinematics, or “high-level control,” governs motion parameters like location, direction, velocity, and acceleration, shaping the desired trajectory. Kinetics, or “low-level control,” related to the control of individual muscles and forces. Multiple trajectories can achieve the same goal-oriented movement, and research has examined how the sensorimotor cortex represents these features (Branco et al., 2019; Zhou et al., 2022). However, how the brain optimally executes voluntary movements remains a major challenge. Ghosh et al. reviewed the neural correlates predictive of upper limb motor intention and kinematics. This review also highlights the potential of closed-loop EEG-based BMIs to promote long-term rehabilitation, neural plasticity, and motor recovery.

In conclusion, the articles in this Research Topic provide insights into the neural mechanisms of motor planning in assisted voluntary movement and advancements in BMI-controlled assistive robotics. However, challenges remain, including the need for larger studies, standardized methodologies, and rigorous bias assessments. Future BMI systems for motor rehabilitation should focus on integrating multiple physiological signals, ensuring long-term stability, improving user engagement, and enhancing sensory feedback. Continued research and clinical trials are essential to developing effective BMI systems and improving the quality of life for patients with motor impairments.
